# Early Initiation of SGLT2 Inhibitors in Acute Heart Failure: a Focus on Diuresis and Renal Protection

**DOI:** 10.1007/s10557-023-07512-6

**Published:** 2023-10-13

**Authors:** Andreas Hammer, Alexander Niessner, Patrick Sulzgruber

**Affiliations:** Department of Internal Medicine II, Division of Cardiology, Medical University of Vienna, Waehringer Guertel 18-20, 1090 Vienna, Austria

**Keywords:** SGLT2 inhibitors, Acute heart failure, Decongestion

## Abstract

**Purpose:**

Acute heart failure (AHF) represents a critical and life-threatening condition characterized by the sudden onset or exacerbation of symptoms, such as dyspnea and fluid retention, due to impaired cardiac function. Despite advances in the treatment of chronic heart failure (HF), the management of AHF remains challenging, with limited therapeutic options available. Sodium-glucose cotransporter 2 (SGLT2) inhibitors have emerged as a promising drug class in AHF management.

**Methods/Results:**

The objective of this article was to conduct a comprehensive review of the existing literature in the domain of SGLT2 inhibitors and their relevance in the context of AHF.

**Conclusion:**

The existing evidence underscores the importance of SGLT2 inhibitors in enhancing decongestive therapy for AHF patients. Early initiation appears both practical and beneficial, leading to improved and sustained decongestion, a reduction in heart failure–related events, enhanced quality of life, and decreased mortality rates, all while maintaining a favorable safety profile. Consequently, it should be considered to initiate SGLT2 inhibitor treatment as early and as safely as possible to facilitate effective decongestion. However, careful patient selection and monitoring are essential when considering the use of these drugs in the management of AHF.

## The Prognostic Value of Decongestion in Acute Heart Failure

Acute heart failure (AHF) is a medical emergency characterized by rapid onset or worsening of symptoms such as shortness of breath, fatigue, and fluid accumulation [1]. It is furthermore a leading cause of hospitalization and death, with a high readmission rate and poor prognosis [1].

As strongly suggested by current guidelines, decongestion, mainly mediated through intensified diuretic therapy, is crucial to restore the euvolemic state [1]. Additionally, to achieve this reduction in excess water, stable renal function is key. Although high doses of loop diuretics are being utilized, the majority of patients are discharged with persistent clinical signs of fluid overload, which is significantly associated with a worse outcome. In this regard, evidence from the Diuretic Optimization Strategies Evaluation (DOSE) trial demonstrated that merely 15% of patients were successfully decongested following 72 h of treatment [2].

Recently, there is a growing interest in the use of sodium-glucose cotransporter 2 (SGLT2) inhibitors in the treatment of AHF, as these drugs have shown promising results in improving cardiovascular outcomes in patients with heart failure (HF) across the entire range of ejection fraction (EF) [3–5]. SGLT2 inhibitors block glucose reabsorption in the proximal tubules of the kidney, resulting in increased urinary glucose and sodium excretion. Given the principle of osmosis water follows the sodium gradient, thus most diuretics act by promoting natriuresis. Under physiologic conditions, the majority of sodium reuptake will be conducted in the proximal tubules (65%), with declining reabsorption rates in later parts of the system (Henle loop 35%; 5% distal tubules) [6]. Since routinely utilized loop diuretics only act in the nephrons region where about two-thirds of sodium has already been reabsorbed, it might be hypothesized that there is missed diuretic potential in the proximal region. Conversely, the SGLT2 is located in the proximal tubules [7]. Interestingly, in acutely decompensated patients, sodium reabsorption shifts even more toward the proximal tubules [6]. Hence, this underlines the importance of additional natriuresis in this specific area in the setting of AHF.

## Early Initiation of SGLT2 Inhibitors for Rapid Decongestion

The optimal time of initiation of SGLT2 inhibitors in the treatment of AHF is crucial but widely discussed. In the clinical trials investigating the use of SGLT2 inhibitors in patients with HF, these drugs were initiated in stable patients with chronic HF. However, there is a growing interest in using SGLT2 inhibitors in the setting of AHF. Consequently, the benefit of SGLT2 inhibitors in patients with AHF has been evaluated in the EMPagliflozin in patients hospitalized with acUte heart faiLure who have been StabilizEd (EMPULSE) trial [8]. This randomized, double-blind, placebo-controlled trial included 530 patients with AHF irrespective of their left ventricular function. In this trial, 10 mg empagliflozin showed a clinical benefit compared to placebo at 90 days for the hierarchical composite endpoint of all-cause death, HF events (HF hospitalization, urgent HF visits, unscheduled HF-related outpatient visits), time to first HF event, and change in baseline Kansas City Cardiomyopathy Questionnaire Total Symptom Score (KCCQ-TSS) (≥ 5 point difference) [8–10]. The empagliflozin group demonstrated a clinical benefit rate of 53.9%, whereas the placebo group displayed a rate of 39.7% (*p* = 0.0054). This translated into notable reductions in mortality (4.2% vs. 8.3%), heart failure events (10.6% vs. 14.7%), and a more pronounced change in KCCQ-TSS from baseline to a 90-day mean of 36.19 vs. 31.73, when compared to the placebo arm [9]. Hence, empagliflozin has also improved symptoms and quality of life measures in patients with AHF. The safety profile of SGLT2 inhibitors in patients with AHF appears to be favorable, with no incident of ketoacidosis and no increase in the risk of adverse renal events [8]. Moreover, acute renal failure was less prominent in the empagliflozin group (7.7% vs. 12.1%), along with fewer rates of urinary tract infections (4.2% vs. 6.4%). These findings suggest that there is clearly a role for early initiation of SGLT2 inhibitors in patients with AHF [8].

SGLT2 inhibitors mediate pleiotropic effects, particularly diuresis and natriuresis, and the reduction of oxidative stress may be relevant in AHF [7]. The SGLT2 inhibitor-related natriuresis in the proximal tubules, in conjunction with not reabsorbed intratubular glucose, may provide considerably improved diuretic volume and earlier decongestion. These effects may be particularly beneficial in the setting of AHF, where rapid removal of excess fluid and reduction of congestion are critical. In this context, during the 90-day follow-up period of EMPULSE, patients treated with empagliflozin demonstrated a notably greater and persistent reduction in congestion (adjusted mean difference of − 1.53 kg, *p* = 0.0137) [8]. This likely represents the causal connection between a lower occurrence of HF events observed in the treatment group, with 26 events compared to 52 [8].

Additionally, evidence from the Effect of Sotagliflozin on Cardiovascular Events in Patients with Type 2 Diabetes Post Worsening Heart Failure (SOLOIST WHF) trial also suggests the benefit of early therapy initiation [11]. This randomized controlled trial demonstrated that sotagliflozin, a dual SGLT1 and 2 inhibitor, led to a significant decrease in the primary endpoint of cardiovascular death, hospitalizations for HF, and urgent visits for HF (HR 0.67, *p* < 0.001) when administered either at the time of patient discharge or within 3 days following discharge [11]. However, the risk reduction was mainly driven by hospitalizations for HF and urgent visits for HF (0.64, *p* < 0.001), since mortality rates did only slightly differ between the groups (10.6% sotagliflozin vs. 12.5 placebo, *p* = 0.36) [11]. This effect was observed, regardless of the patients’ ejection fraction. Furthermore, the treatment arm demonstrated a notable improvement in quality of life as measured by the mean change in KCCQ-12 score at month 4, with scores favoring the treatment group (17.7 vs. 13.6). It may be hypothesized that only the SGLT2 component of the drug contributed to better decongestion since SGLT1 is mainly expressed within the gastrointestinal tract and inhibition of it results in reduced postprandial glucose levels [11].

Thus, SGLT2 inhibitors have emerged as a promising class of drugs in the treatment of AHF, since these drugs have shown to improve cardiovascular outcomes, reduce HF hospitalizations, and improve symptoms and quality of life measures in patients with AHF.

## Preserving Kidney Function During Diuretic Therapy

The effectiveness of diuretic regimens involving loop diuretics in individuals with acute decompensated heart failure is frequently hindered by the occurrence of declining kidney function. The single-center, randomized study Empagliflozin in Acute Decompensated Heart Failure (EMPAG-HF) evaluated the role of empagliflozin (25 mg o.d.) in addition to standard decongestive therapy in patients with acute decompensated HF in regard to urine output over 5 days. They observed that empagliflozin-treated patients showed a 25% increase in 5-day urine output compared to standard treatment (median 10,775 mL vs. 8650 mL) [12]. This effect was facilitated without deterioration of renal function (estimated glomerular filtration rate (eGFR), 51 ± 19 vs. 54 ± 17 mL/min per 1.73 m^2^; *p* = 0.599) nor kidney injury [12]. Additionally, a greater decline in NT-proBNP levels was found among empagliflozin receivers after 5 days (− 1861 vs. − 727.2 pg/mL; *p* < 0.001) [12]. Metra et al. also emphasized the significance of preserving kidney function in patients with residual congestion after hospital discharge [13]. They found that worsening kidney function alone, defined as an increase in creatinine levels of ≥ 0.3 mg/dL, did not have prognostic value for adverse outcomes [13]. However, the combination of elevated creatinine levels and persistent signs of congestion at discharge independently predicted a higher risk of death or death/rehospitalization, underscoring the importance of this combined association in predicting adverse outcomes [13].

## When to Initiate SGLT2 Inhibitors in Acute Heart Failure?

Within the EMPULSE trial, patients were initially stabilized and subsequently randomized; hence, SGLT2 inhibitor therapy was established in a median duration of 72 h, whereas, in the EMPAG-HF trial, SGLT2 inhibitors were introduced within a 12-h timeframe after hospital admission [12]. It is worth noting that in EMPAG-HF, patients with an eGFR below 30 mL/min/1.73 m^2^ or those requiring hemofiltration or any other form of extracorporeal therapy were excluded, which differed from the exclusion criteria in EMPULSE [12]. Furthermore, patients with AHF attributed to any etiology causing HF decompensation necessitating immediate intervention, such as acute coronary syndrome, unstable arrhythmias, mechanical abnormalities, or acute pulmonary embolism, were excluded from both studies [12, 8].

Nevertheless, it is essential to recognize that early initiation of SGLT2 inhibitors in AHF carries potential risks, such as the occurrence of ketoacidosis, particularly in patients with elevated lactate levels, and those with elevated inflammatory markers indicative of an infectious state. These risks should be approached cautiously. However, once the HF medication is initiated or resumed, the inclusion of SGLT2 inhibitors in the therapeutic regimen is important. In this context, findings from the EMPAG-HF trial demonstrate the safety and tolerability of initiating SGLT2 inhibitors early, with no significant differences observed compared to the placebo group [12].

Recent findings, as emphasized by the Acetazolamide in Decompensated Heart Failure with Volume Overload (ADVOR) trial, have indicated that the addition of the carbonic anhydrase inhibitor acetazolamide to standard therapy leads to a higher rate of successful decongestion in patients experiencing acute decompensated HF [14]. It is important to note that recipients of SGLT2 inhibitors were excluded from participation in this trial. Given that both drugs act to reduce sodium reabsorption in the proximal tubules, although via different mechanisms, the potential for synergistic or adverse effects combining these substances remains uncertain. As such, it is currently a subject of speculation whether the addition of SGLT2 inhibitors to acetazolamide therapy yields additional benefits in terms of decongestion.

In conclusion, SGLT2 inhibitors have demonstrated their value in augmenting decongestive therapy in AHF patients. The existing evidence from the EMPULSE and EMPAG-HF trials strongly supports the beneficial effects linked to early initiation of empagliflozin in AHF patients. Consequently, it should be considered to initiate SGLT2 inhibitor treatment as early and as safely as possible to facilitate prompt and sustained decongestion. However, careful patient selection and monitoring are essential when considering the use of these drugs in the management of AHF.

## Data Availability

Not applicable.
